# Synergism between upregulation of Rab7 and inhibition of autophagic degradation caused by mycoplasma facilitates intracellular mycoplasma infection

**DOI:** 10.3892/mmr.2014.1907

**Published:** 2014-01-20

**Authors:** XIAOPENG HU, JIE YU, XIANG ZHOU, ZHAOMING LI, YUN XIA, ZHIYONG LUO, YAQUN WU

**Affiliations:** 1 Department of General Surgery, Tongji Hospital, Tongji Medical College, Huazhong University of Science and Technology, Wuhan, Hubei 430030, P.R. China; 2 First People’s Hospital of Jiujiang City, Jiujiang, Jiangxi 330300, P.R. China; 3 The Fifth Hospital of Huangshi City, Huangshi, Hubei 435004, P.R. China; 4 Department of Oncology, The First Affiliated Hospital, Zhengzhou University, Zhengzhou, Henan 450052, P.R. China

**Keywords:** mycoplasma, endocytic pathway, autophagy, Rab7, LC3, p62

## Abstract

Following fusion of a mycoplasma with a host cell membrane, the inserted components of mycoplasma may then be transported through the endocytic pathway. However, the effects of mycoplasmas on the host cell endomembrane system are largely unknown. In this study, mycoplasma-induced changes in the dynamics of endocytic and autophagic systems were investigated. Endocytosis and autophagy are two major processes involved in the survival of intracellular prokaryotic pathogens. It was found that, immediately following infection, mycoplasmas induce endocytosis in the host cell; however, in the long term the mycoplasmas suppress turnover of the components of the endocytic pathway. Immunofluorescence microscopy revealed that Rab7 and LC3-II are recruited to the intracellular mycoplasma-containing compartments. Western blot analysis and quantitative reverse transcription-polymerase chain reaction (qPCR) showed that mycoplasmas increase expression of Rab7 by upregulating transcription, but increase levels of LC3-II and p62 by post-translational regulation. Furthermore, it was demonstrated that mycoplasma infection causes inhibition of autophagic degradation of LC3-II and p62. In addition, it was found that upregulation of Rab7 and inhibition of autophagic degradation synergistically contributes to intracellular mycoplasma accumulation. In conclusion, these findings suggest that mycoplasmas may manipulate host cell endosomal and autophagic systems in order to facilitate intracellular infection.

## Introduction

The lack of a cell wall renders mycoplasmas readily able to fuse with host cell membranes (1). The consequences of this remain largely unknown, since the fusion results in the insertion of various mycoplasmal cell components, complicating the interpretation of the pathogenesis (2–4). The fusion process has been observed using transmission electron microcopy (5). Following fusion with host plasma membrane or endosomal membrane, the mycoplasma membrane components integrate into the host cell membrane, along with membrane-associated proteins (1). These membrane-associated proteins are subsequently transported through the endocytic pathway.

The endocytic pathway is a highly dynamic membrane system consisting of a heterogeneous population of early, recycling and late endosomes. The endosomes receive the endocytosed membrane-bound vesicles and sort them for maturation, recycling or lysosomal degradation, respectively. It is currently proposed that the trafficking pathway of the endosomal system comprises three major processes: i) Constitutive recycling of early endosomes or intermediate endosomes to the plasma membrane; ii) fusion of multivesicular bodies (MVBs) with lysosomes for lysosomal degradation; and iii) fusion of MVBs with plasma membrane for exosomal release of intraluminal vesicles (6). All of these processes are crucial for cells to perform their normal physiological functions (7–9).

MVBs are morphologically distinct late endosomes that originate from an endosomal compartment through membrane invagination and the formation of intraluminal vesicles ranging between 50 and 100 nm in diameter (10). Prevention of the fusion between MVBs and lysosomes results in the inhibition of lysosomal degradation (9). The autophagic pathway, another evolutionarily conserved membrane system with pleiotropic functions, has a role in the cellular response to intracellular infection by which host cells eliminate invading microorganisms (11). MVBs and autophagosomes fuse prior to the lysosomal fusion step to form amphisomes (12). The endosomal system provides membrane components for autophagosome membrane biogenesis (13) and the amphisomes acquire machinery required for lysosomal fusion following the fusion of nascent autophagosomes with endosomes (14). The autophagosomal system overlaps extensively with the endosomal system and the autophagosome-lysosome fusion is a process that recapitulates endosome-lysosome fusion. Therefore, measuring the turnover of the substrates of autophagic degradation may partly reflect the rate of endolysosomal degradation.

Host cellular molecules, such as LC3, p62 and Rab7, are involved in the aforementioned processes of autophagic degradation. LC3-I undergoes post-translational modification to form lipidated LC3-II, an autophagosomal marker. p62 (SQTSM1), is an adaptor of LC3-II and serves as a substrate for autophagic degradation (15). Rab7 small GTPase contributes to the progression of the autophagy pathway and regulates trafficking of MVBs to lysosomes (16,17). Numerous pathogenic microorganisms are capable of subverting host cellular endosomal and autophagosomal pathways in order to facilitate intracellular infection, often involving host cellular Rab family GTPases and LC3-II-positive vacuoles (18). For example, *Mycoplasma pneumonia* toxin induces the formation of vacuoles in host cells (19), which is associated with Rab GTPase functions. However, little is known about mycoplasma-induced modifications in the dynamics of the host cellular endomembrane system, or the host molecules involved in intracellular mycoplasma infection.

In this study, the effects of mycoplasma infection on the host cell endosomal and autophagosomal systems were investigated, as well as the molecular basis of intracellular mycoplasma accumulation involving Rab7 and autophagic markers LC3-II and p62.

## Materials and methods

### Antibodies and reagents

The primary antibodies used were as follows: LC3B (3868; Cell Signaling Technology, Inc. Beverly, MA, USA); p62/Sqstm1 (AT4033a; Abgent, San Diego, CA, USA); and Rab7 (55469-1-AP; Proteintech Group, Chicago, IL, USA). The secondary antibodies were obtained from Jiayuan Biotech (Wuhan, China). DiI was obtained from Molecular Probes (Carlsbad, CA, USA).

### Cell culture

HeLa and SH-SY5Y cells were obtained from the American Type Culture Collection (Manassas, VA, USA) and maintained in Dulbecco’s modified Eagle’s medium (DMEM, high glucose; Hyclone, Logan, UT, USA) and a 1:1 ratio of DMEM/F12 medium (Gibco-BRL, Carlsbad, CA, USA), respectively. Culture medium was supplemented with 10% fetal bovine serum (FBS; Hyclone) and 2 mM L-glutamate prior to use. The cells were cultured at 37ºC in a humidified 5% CO_2_ atmosphere.

### Mycoplasma identification

The species of mycoplasma used were identified using polymerase chain reaction (PCR) with mycoplasma genus-specific degenerate primers, and followed by agarose gel electrophoresis, purification and T-vector cloning (pMD-18T vector, Takara, Dalian, China). The sequencing results confirmed that the mycoplasma contaminant was identical to *Mycoplasma pulmonis (M. pulmonis)* UAB CTIP strain (NC_002771.1). The degenerate primers used for the conserved 16s-23s gene spacer region of the mycoplasma genus were as follows: forward 5′-ACACCATGGGAGYTGGTAAT-3′, reverse 5′-CTTCWTCGACTTYCAGACCCAAGGCAT-3′.

### Preparation of mycoplasma-containing cell culture supernatant

HeLa cells contaminated with *Mycoplasma pulmonis* were plated at a density of 1×10^5^ cells/ml in a six-well plate. The next day, the cells were washed three times with 0.01 M phosphate-buffered saline (PBS) and incubated with 2 ml DMEM without FBS for 24 h. The cell culture supernatant was collected and cell debris was removed by filtration through a 0.8-μm cut-off filter (Millipore, Bedford, MA, USA).

### Quantitative PCR (qPCR) for mycoplasma and host cell genome copy numbers

For absolute qPCR, the pMD-18T plasmid (mycoplasmal sequence inserted) was diluted in a 1:10 serial dilution five times and utilized as the standard template The absolute copy number of mycoplasmas in the mycoplasma-containing cell culture supernatant was determined by comparison with the standard curve, and the multiplicity of infection (MOI) was determined accordingly.

For relative qPCR, the intracellular mycoplasma copy numbers were normalized to host genome copy numbers. The host cells were suspended in 0.01 M PBS and washed five times to remove extracellular planktonic mycoplasma. The genomic DNA was then extracted from total cell extracts and purified as previously described (20).

qPCR was performed using the LightCycler^®^ 480 Real-Time PCR Detection System (Roche, Basel, Switzerland) and an SYBR Green Real-Time PCR 2× premix kit (Takara). The reaction conditions were as follows: 15 sec at 95ºC followed by 45 cycles of denaturation for 20 sec at 95ºC and annealing and extension for 20 sec at 60ºC. The melting curve of the products was determined and found to be specific. Primers used for the *M. pulmonis* 16s–23s gene spacer region were as follows: forward, 5′-GGAGCTGGTAATGCC CAAAGT-3′ and reverse, 5′-ACGTTCTCGTAGGGATAC CTTG-3′. Primers for host cell genome (β-hemoglobin) were as follows: forward, 5′-GAAGAGCCAAGGACAGGTAC-3′ and reverse, 5′-CCAACTTCATCCACGTT CAC-3′.

### qPCR

Total RNA was extracted from HeLa cells using TRIzol (Invitrogen, Carlsbad, CA, USA), and first strand cDNA reverse transcription was performed using a reverse-transcription RT-PCR kit (Takara) in the presence of Oligo (dT) 15 primers. qPCR was conducted using a LightCycler® 480 Real-Time PCR Detection System (Roche) and SYBR Green Real-Time PCR 2× premix kit (Takara). The reactions were performed under the following conditions: 15 sec at 95ºC followed by 40 cycles of denaturation for 15 sec at 95ºC and annealing and extension for 15 sec at 60ºC. The melting curves for Rab7, LC3 and p62 were determined and found to be specific. A two standard curve method was used to quantify the expression levels of Rab7, LC3 and p62, and the mRNA levels were normalized to that of β-actin. Primers used for Rab7, LC3, p62 and β-actin were as follows: Rab7 forward 5′-CATCCTGGGAGATTCTGGAGTC-3′ and reverse, 5′-TGT GTCCCATATCTGCATTGTG-3′; LC3 forward, 5′-GAGCAG CATCCAACCAAAATC-3′ and reverse, 5′-GCCTGATTA GCATTGAGCTGTAAG-3′; p62 forward, 5′-GGACTTGGT TGCCTTTTCCAGTG-3′ and reverse, 5′-GCAGCCGTC GCAGATCACAT-3′; β-actin forward, 5′-GCACCCAGC ACAATGAAGATC-3′ and reverse, 5′-CTCGTCATACTC CTGCTTGCTG-3′.

### Fluorescence microscopy

HeLa cells were plated on 0.17-mm coverslips or glass-bottomed Petri dishes for fluorescence microscopy. Images were captured using an Olympus FluoView™ FV1000 confocal microscope (100×/1.40 oil lens) or an Olympus BX51 wide field upright microscope with a 60× 1.4 NA objective. DiI was used to label the cell membrane at 2 μM for 15 min at 37ºC. HeLa cells expressing green fluorescent protein-tagged Rab7 (GFP-Rab7) were fixed by 4% paraformaldehyde (PFA) at room temperature for 10 min, prior to the cells being observed by confocal microscopy. DAPI (300 μM) was used to counterstain the nuclei for 10 min at room temperature. For immunostaining of Rab7 and LC3-II, HeLa cells were fixed by 4% PFA and permeabilized by 0.1% Triton-X100 followed by blocking in 3% bovine serum albumin (BSA). The cells were then incubated with Rab7 and LC3B primary antibodies at 4ºC overnight in a humidified dark chamber. The following day, Alexa Fluor 488-labeled secondary antibodies were used to visualize the staining.

### Western blot analysis

Western blot analyses were performed as previously described (21). For immunoblotting of LC3 in SH-SY5Y cells, mycoplasma-infected (infected at the MOI ratio of 10:1 for 24 h) cells and control SH-SY5Y cells were serum starved for 2 h prior to treatment with 0.1% dimethylsulfoxide (DMSO) or Bafilomycin A1 (Baf A1; 10 nM) for 4 h; and lysates were collected for western blot analysis against LC3. For western blot analysis of p62 levels, the cellular protein translation was blocked with 35 μM cycloheximide (CHX) for 4, 8 or 12 h. The densitometry was analyzed using ImageJ software (Rasband WS, US National Institutes of Health, Bethesda, MD, USA). The densitometric ratio of LC3-II to β-actin band was expressed as fold-change relative to control cells treated with DMSO.

### Plasmids, small interfering RNA (siRNA) and transfection

Human wild-type Rab7 coding sequence was cloned and inserted into *Eco*RI/*Bam*HI site of pEGFP-C1 vector downstream of the GFP sequence in the right reading frame. siRNA duplexes were purchased from RiboBio Co., Ltd. (Guangzhou, China). HeLa cells were transfected with plasmids or siRNA duplexes (10 nM) using Lipofectamine™ 2000 (Invitrogen) in accordance with the manufacturer’s instructions. Rab7 and scrambled control siRNA duplexes were as described previously by Vanlandingham and Ceresa (16) with the modifications of two deoxythymidine added to the 3′ end of each sequence to make it more stable.

### Statistical analysis

Data from three independent experiments are expressed as mean ± standard deviation (SD). The Student’s t-test was used for comparisons between different groups. In all cases, P<0.05 was considered to indicate a statistically significant difference.

## Results

### Mycoplasmas affect the host cell endosomal system, promoting endocytosis and suppressing turnover of endocytic membranes

Mycoplasma membrane components are inserted into host cell membranes and endocytosed; as a result, mycoplasmas may interfere with host cell through the endocytic pathway. Therefore, changes elicited by mycoplasmas in the host cell endosomal system were investigated in this study.

**Figure 1:**
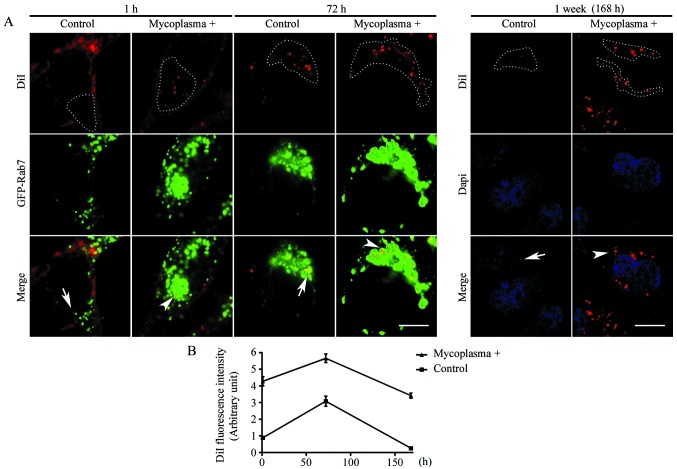
Mycoplasma infection affects the dynamics of the host cell endosomal system. (A) Cell culture supernatants from control and mycoplasma-positive HeLa cells were prepared as described in the Materials and methods. Mycoplasma-free HeLa cells transfected with green fluorescent protein-tagged Rab7 (GFP-Rab7) plasmids were labeled with membrane lipid dye DiI (2 μM) for 15 min followed by incubation with the control or the mycoplasma-positive cell culture supernatant for 45 min. One hour following DiI labeling, the HeLa cells expressing GFP-Rab7 were observed by confocal microscopy. The cells treated in parallel were maintained in regular growth media until the observation at 72 h and one week after labeling. As the transient expression of GFP-Rab7 is not able to be observed in the long-term culture, DAPI staining was used to visualize the nucleus for observation at one week. The region of interest (ROI) for each cell is delineated by the white dotted line. The white arrows and arrow heads indicate the DiI-labeled vesicles in control and in mycoplasma-contaminated HeLa cells, respectively. The DiI-labeled vesicles were observed within the lumen of the Rab7-positive vacuoles at 72 h and persisted in the mycoplasma-contaminated HeLa cells for more than one week. The scale bar is 10 μm. (B) The DiI fluorescence intensity per cell within the ROI (observed at 1 h, 72 h and 1 week) was analyzed using ImageJ software (Rasband WS, US National Institutes of Health, Bethesda, MD, USA). The plotted data were from ≥30 cells from three independent experiments. The error bar represents the mean ± standard deviation.

In mycoplasma-infected HeLa cells, it was observed that the GFP-Rab7 vesicles accumulated in the peri-nuclear region 1 h post-infection with the mycoplasma, and that the GFP-Rab7 vesicles were increased in size compared with control cells (Fig. 1A). These morphological changes in late endosomal compartments indicate that the dynamics of the endosomal system were subverted by mycoplasma infection.

In order to investigate the mycoplasma-induced alterations in the dynamics of the endosomal system, chase assays for endocytosed membrane components in mycoplasma-infected HeLa cells and control cells were performed separately. Due to the fact that DiI is able to be embedded into lipid bilayers, and may be utilized as a marker for studying membrane orientation and fusion patterns, DiI was used to label the cell membrane for observation (22,23). One hour after labeling, the endocytosed DiI-stained plasma membrane components were observed to have merged with the surrounding membrane of Rab7-positive compartments, which reflected the docking and/or fusing sites of DiI-stained endocytic vesicles with Rab7-positive late endosomes (Fig. 1A). However, after 72 h, the DiI-stained vesicular structures were present in the lumen of Rab7-positive late endosomes. This phenomenon was more prominent in mycoplasma-infected cells compared with non-infected cells (Fig. 1A).

In mycoplasma-infected HeLa cells, the average fluorescence intensity of the DiI-stained endocytic vesicles within the peri-nuclear region (region of interest, ROI) per cell was four-fold higher than that in the control cells (Fig. 1B). In a prolonged observation, the membrane lipid dye that was transferred through the endocytic pathway persisted within the Rab7-positive compartments in the mycoplasma-infected HeLa cells with higher intensity relative to that in the control cells (Fig. 1B). The intracellular persistence of the DiI-bearing endosomal structures suggest that the elimination of endocytosed membrane components is suppressed in mycoplasma-infected HeLa cells.

In summary, the increase in the size of the endocytic vesicles indicates that mycoplasma may promote endocytosis immediately post-infection. The increased number and intensity of the DiI-bearing endocytic structures also indicates that mycoplasma infection suppresses turnover of the endocytosed membrane components in HeLa cells.

### Mycoplasma infection upregulates Rab7 but appears to block the degradation of LC3-II and p62

**Figure 2:**
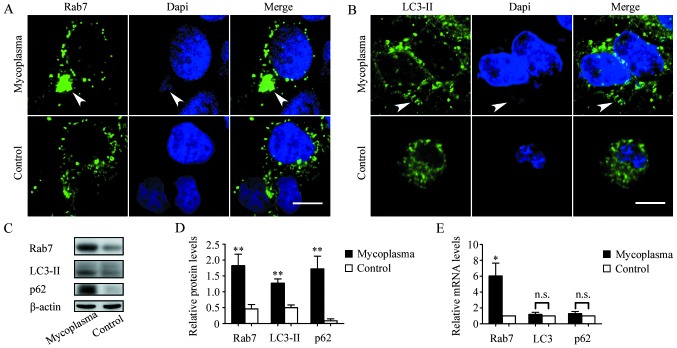
Effects of mycoplasma infection on the regulation of Rab7, LC3 and p62. HeLa cells contaminated with *Mycoplasma pulmonis* in long-term culture were immunostained for (A) Rab7 or (B) LC3-II and observed by confocal microscopy. The white arrow head indicates the extranuclear DAPI staining where endogenous Rab7 and LC3-II were recruited to the inclusion-like bodies. The scale bar is 10 μm. (C) HeLa cells contaminated with or without *Mycoplasma pulmonis* were harvested and subjected to western blot analysis for Rab7, LC3-II and p62. In addition, β-actin was used as a loading control. One representative result from three independent experiments is shown. (D) Relative protein levels of Rab7, LC3-II and p62, which are normalized to the densitometry of β-actin bands, from three independent experiments compared with control (^**^P<0.01). (E) Relative mRNA levels of Rab7, LC3 and p62, which are normalized to the mRNA levels of β-actin, from three independent experiments compared with control (^*^P<0.05). Error bars represent the mean ± standard deviation (SD). n.s., not significant.

Intracellular infection is associated with host cellular Rab7 and LC3, therefore, whether endogenous Rab7 and LC3-II were recruited to the surface of the intracellular mycoplasma-containing compartment was investigated. HeLa cells infected with *M. pulmonis* were immunostained for Rab7 and LC3-II. Confocal images revealed that, in mycoplasma-infected HeLa cells, the extranuclear DAPI-positive particles were surrounded by membrane structures that were positively stained for Rab7 and LC3-II, and which were not observed in the negative controls (Fig. 2A and B). In addition, the fluorescence intensity of the endogenous Rab7 vesicles and LC3-II puncta/cell was higher in the mycoplasma-infected HeLa cells (data not shown). Therefore, Rab7 and LC3-II protein levels were investigated in HeLa cells contaminated with or without *M. pulmonis*. The levels of Rab7 and LC3-II were found to be upregulated in mycoplasma-infected HeLa cells compared with the negative control cells (Fig. 2C and D). The increased level of LC3-II in HeLa cells infected with *M. pulmonis* may result from either induction of autophagy or inhibition of lysosomal degradation; however, the accumulation of p62 (SQSTM1), which serves as a cargo adaptor for autophagic degradation (24,25), indicated that it was the degradation process that was blocked in mycoplasma-infected HeLa cells (Fig. 2C and D). The expression levels of Rab7, LC3 and p62 mRNA were also measured using qPCR. The results showed that mRNA expression of Rab7 was upregulated following mycoplasma infection, whereas the mRNA expression of LC3 and p62 remained unchanged (Fig. 2E).

### Mycoplasma infection results in inhibition of autophagic degradation

**Figure 3:**
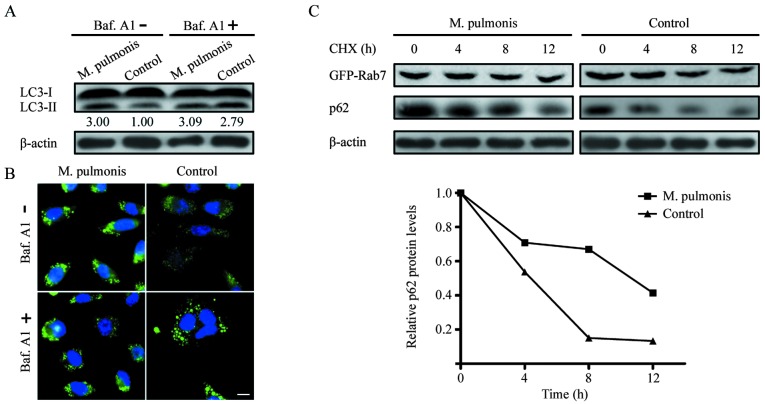
Mycoplasma infection suppresses autophagic degradation of LC3-II and p62. (A) SH-SY5Y cells were cultured with or without *M. pulmonis*. The cells were serum-starved for 2 h and then treated with or without 10 nM Baf A1 for 4 h prior to harvest. Western blot analysis against LC3 was performed on the cell lysates. One representative result from three independent experiments is shown. The numbers below the band represent the relative densitometric ratios of LC3-II/β-actin, which was set to 1 in the control cell treated without Baf A1. (B) The cells treated in parallel to (A) were immunostained for LC3-II puncta and observed by a wide field fluorescent microscope (the scale bar represents 10 μm). (C) Mycoplasma-free HeLa cells were transfected with GFP-Rab7 plasmid, 6 h after the transfection the cells were infected with or without *M. pulmonis* (MOI 10:1) for an additional 24 h. Then the cells were treated with or without 35 μM CHX for 4, 8 or 12 h. The relative levels of p62 (densitometric ratio of p62/β-actin) were identified by western blot analysis. The initial ratio at time zero was set to 1. One representative result from three independent experiments is shown. GFP, green fluorescent protein; MOI, multiplicity of infection; CHX, cycloheximide; Baf A1, Bafilomycin A1.

LC3-I is difficult to detect in HeLa cells by immunoblotting; therefore, SH-SY5Y cells, which express high levels of LC3-I, were used to investigate the alteration in LC3-II induced by *M. pulmonis* infection in the presence or absence of Baf A1. Prior to adding Baf A1, the cells were serum-starved to elicit an increase in autophagy. The results revealed that, in the mycoplasma-free cells, Baf A1 caused ~2.8-fold increase in the LC3-II level, whereas in the mycoplasma-infected cells only a slight increase in LC3-II level was observed (Fig. 3A), indicating that lysosomal degradation of LC3-II is blocked in mycoplasma-infected cells. Furthermore, in the presence of Baf A1, little difference was observed in LC3-II levels between mycoplasma-free and mycoplasma-infected cells (Fig. 3A), suggesting that induction of autophagy is not subverted by mycoplasma infection. These findings suggest that the mycoplasma-induced increase in LC3-II level was mainly due to the inhibition of autophagic degradation of LC3-II. In addition, observation of the LC3-II puncta by fluorescence microscopy revealed a similar pattern compared with the results of the western blot analysis (Fig. 3B). The degradation of p62 was also investigated using western blot analysis in a cycloheximide (CHX) chase assay in HeLa cells. Rab7 was found to be upregulated in mycoplasma-infected HeLa cells, which may have contributed to lysosomal degradation of p62 (17); therefore wild-type GFP-Rab7 was overexpressed in HeLa cells with or without mycoplasma infection to avoid bias. The results revealed that the degradation of p62 was inhibited in mycoplasma-infected HeLa cells, which was not rescued by overexpression of Rab7 (Fig. 3C).

### Synergism between upregulation of Rab7 and inhibition of autophagic degradation is conducive to intracellular accumulation of mycoplasmas

**Figure 4:**
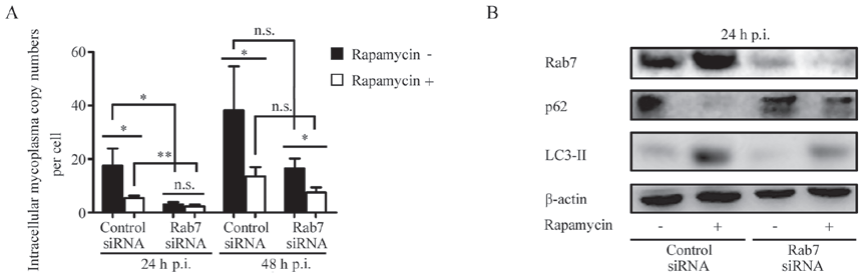
Upregulation of Rab7 combined with inhibition of autophagic degradation synergistically facilitate intracellular accumulation of mycoplasmas. (A) HeLa cells were transfected with Rab7 or control siRNA for 36 h followed by incubation with or without 100 nM rapamycin for 12 h. Rab7-depleted and control cells were then infected with cell culture supernatent containing *M. pulmonis* at an MOI of 10:1. The intracellular mycoplasma DNA copy numbers were determined using quantitative PCR at 24 and 48 h post-infection and normalized against the copy numbers of the host cell genome (n=3, t-test). ^*^P<0.05, ^**^P<0.01. (B) HeLa cells treated as in (A) were lysed at 24 h post-infection and subjected to western blot analysis with indicated antibodies. One representative result from three independent experiments is shown. siRNA, small interferring RNA; MOI, multiplicity of infection; PCR, polymerase chain reaction; p.i., post-infection. n.s., not significant.

Rab7 is involved in the intracellular infection of microorganisms (18); therefore, the effect of Rab7 on the intracellular accumulation of *M. pulmonis* was investigated. The results revealed that *M. pulmonis* DNA copies accumulated in HeLa cells at 24 and 48 h post-infection, and depletion of Rab7 by siRNA decreased the intracellular mycoplasma DNA copy numbers (Fig. 4A). Induction of autophagy by Rapamycin also attenuated the intracellular accumulation of *M. pulmonis* (Fig. 4A). Western blot analysis was utilized to confirm the efficiency of Rab7 knockdown in mycoplasma-infected HeLa cells 24 h post-infection (Fig. 4B).

## Discussion

In this study, the effects of mycoplasma infection on the host cell endosomal system were investigated. In particular, mycoplasma-induced changes in the dynamics of endosomal membrane system, as well as in the autophagic degradation process, were studied. These are the two major processes involved in the survival of intracellular prokaryotic pathogens (26).

Following mycoplasma infection, Rab7-positive vacuoles were demonstrated to accumulate in the peri-nuclear region of host cells in the form of large spacious aggregates, which reflected the mycoplasma-induced alterations in the dynamics of the late endosomal system. The accumulation of large spacious late endosomes has also been observed in Salmonella infection, as well as in other microbial infections (18,27). It was hypothesized that mycoplasmas either promote the formation or inhibit the turnover of late endosomes, resulting in the formation of Rab7-positive large, spacious aggregates. Furthermore, semi-quantitative observation of DiI-bearing endocytic vesicles revealed that in mycoplasma-infected host cells the rate of internalization of plasma membrane is enhanced immediately following mycoplasma infection, and that the endocytosed vesicular structures are more resistant to mechanisms of elimination in a long-term culture. These findings suggest that mycoplasma infection inhibits the dynamic turnover of the endosomal system and increases endocytosis of the plasma membrane.

The net effect of mycoplasma-induced alterations in the dynamics of late endosomal turnover results in inhibition of downstream elimination of endocytic membrane components; however, whether the lysosomal degradation and exosomal discharge pathways are involved in the inhibition has yet to be elucidated.

The lysosomal degradation pathway, which involves the maturation of endosomes or autophagosomes followed by fusion with lysosomes, was studied and the role of the lysosomal pathway in mycoplasma-induced cellular responses was investigated. It was found that Rab7 and LC3-II were recruited to the mycoplasma-containing compartments, demonstrating an overlap between the endosomal and autophagosomal pathways during the cellular response to mycoplasma invasion. Western blot analysis, in combination with qPCR, revealed that Rab7 was transcriptionally upregulated, while LC3-II, as well as p62, were upregulated via post-translational mechanisms. Post-translational upregulation of LC3-II and p62 protein levels may result from inhibition of the autophagic flux (15,24). It was therefore hypothesized that the lysosomal degradation process was suppressed by mycoplasma infection, resulting in the accumulation of the autophagosome marker LC3-II and the cargo protein p62, which explains the observed inhibition of endocytic membrane elimination.

The drug Baf A1 is used as a tool to block the autophagic degradation process (28). Using Baf A1 to block autophagic flux, it was found that inhibition in autophagic degradation was the main cause for mycoplasma-induced LC3-II accumulation. Following inhibition of protein synthesis by administration of CHX, p62 proteins levels were chased. Rab7 contributes to maturation of late autophagic vacuoles (29), i.e., the fusion between autophagosomes and lysosomes, which facilitates the degradation of cargo proteins. However, the Rab7 protein expression levels are decreased in mycoplasma-free cells compared with mycoplasma-infected cells. Therefore, in this experiment, Rab7 was overexpressed to eliminate the influence of insufficient Rab7 levels on autophagic degradation of p62. Of note, it was found that mycoplasma infection suppressed cellular p62 degradation, despite the overexpression of Rab7, suggesting that fusion of autophagosomes with lysosome may be impeded by intracellular mycoplasma infection. This hypothesis may also apply to amphisome fusion with lysosomes, since it is also a late autophagic compartment resulting from autophagosome-endosome fusion (12).

In order to investigate the strategy used by the pathogen to manipulate the host cell for intracellular survival, the molecular basis underlying the intracellular accumulation of mycoplasmas was investigated. Rab7 was found to be required for the intracellular accumulation of mycoplasmas and it was revealed that the induction of autophagy contributes to reducing the intracellular mycoplasma accumulation. Upregulation of Rab7 may promote homotypic fusion between Rab7-positive compartments (16), which accounts for the formation of large spacious aggregates of Rab7-positive vacuoles. Therefore, it was hypothesized that the large spacious vacuoles, combined with the inhibition of autophagic degradation, may provide an niche for intracellular pathogens, thereby facilitating intracellular infection of mycoplasmas. Of note, DNA copy numbers were used to indicate that intracellular mycoplasma accumulation occurs regardless of the viability of mycoplasma. The observed increase in the intracellular accumulation of mycoplasmas may also be the result of increased endocytosis of mycoplasmas of extracellular origin rather than from intracellular replication; either explanationsappears consistent with our hypothesis.

In conclusion, mycoplasma infection interferes with the host endosomal system and perturbs the dynamics of the endosomes and autophagosomes by upregulating Rab7, as well as by suppressing autophagolysosomal degradation, resulting in an intracellular niche that may be exploited by mycoplasma or even other intracellular pathogens.

Phagolysosomal maturation arrest caused by intracellular infection of *Mycobacterium tuberculosis* may interfere with inward budding of human immunodeficiency virus (HIV) into multivesicular bodies (30). Similarly, the mycoplasma-induced alterations in the dynamics of the endosomal system may be involved in the pathogenic synergism of mycoplasma with other co-infecting pathogens. Moreover, Rab7, which may be upregulated by mycoplasma infection, is associated with entry of certain enveloped viruses into host cells (31,32). Since other pathogens may also benefit from mycoplasma-induced alterations in the endosomal system, mycoplasmas may serve as helpers or cofactors for other co-infections of clinical significances via manipulation of the host cellular endosomal pathway, involving HIV and *Mycobacterium tuberculosis*.
